# Supplementary material for: Coordinated wound responses in a regenerative animal-algal holobiont
Source: Nat Commun. 2024 May 13;15:4032. doi: 10.1038/s41467-024-48366-2 (PMC11091193; doi:10.1038/s41467-024-48366-2)
Supplement: Supplementary file 1 — Supplementary Information [file 41467_2024_48366_MOESM1_ESM.pdf]

Supplementary Information for

Coordinated wound responses in a regenerative animal-algal holobiont

Dania Nanes Sarfati[1], Yuan Xue[2], Eun Sun Song[3], Ashley Byrne[4], Daniel Le[4], Spyros Darmanis[4], Stephen R. Quake[2,3], Adrien Burlacot[1,5], James Sikes[6,*], Bo Wang[2,7,*]

[1] Department of Biology, [2] Department of Bioengineering, [3] Department of Applied Physics, Stanford University, USA

[4] Chan Zuckerberg Biohub, San Francisco, CA, USA

[5] Department of Plant Biology, Carnegie Institution for Science, Stanford, USA

[6] Department of Biology, University of San Francisco, San Francisco, CA, USA

[7] Department of Developmental Biology, Stanford University School of Medicine, USA

Supplementary Figures & legends

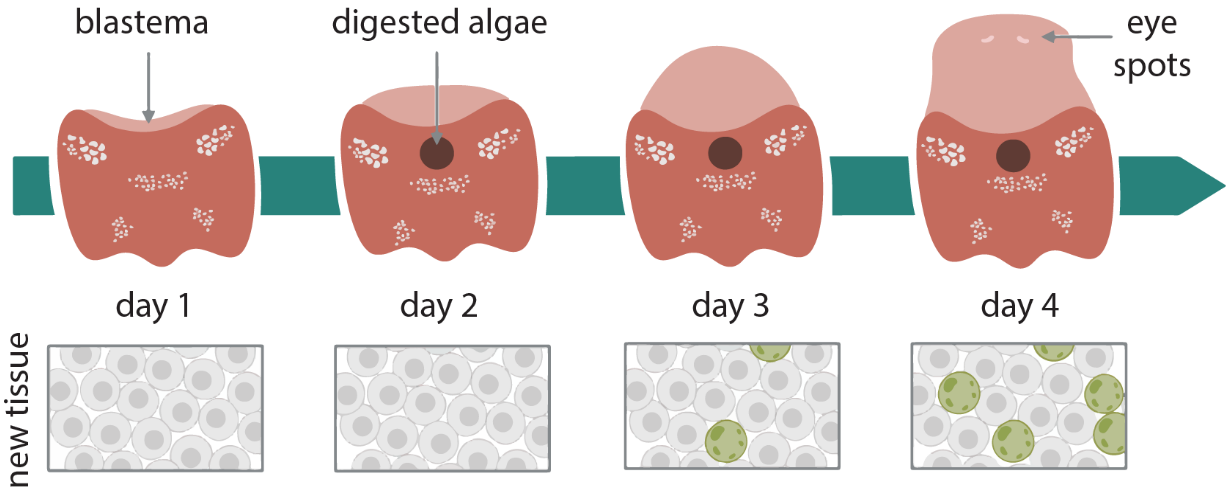

Supplementary Figure 1: Schematic showing the process of anterior regeneration from *C. longifissura* tail fragments.
Animals are bisected at the posterior pair of white concrement granules, which coincides with the fission plane during asexual reproduction. Lighter red depicts the new tissue, with darker color representing the pre-existing tissue. A black spot often forms at 2 dpa, which has been suggested to be the accumulation of digested algae [28,29]. Lower panels: algal population in the new tissue. Algae (green) begin to populate the new tissue at 3 dpa and completely cover it by 4 dpa. Schematics are created with BioRender.com.

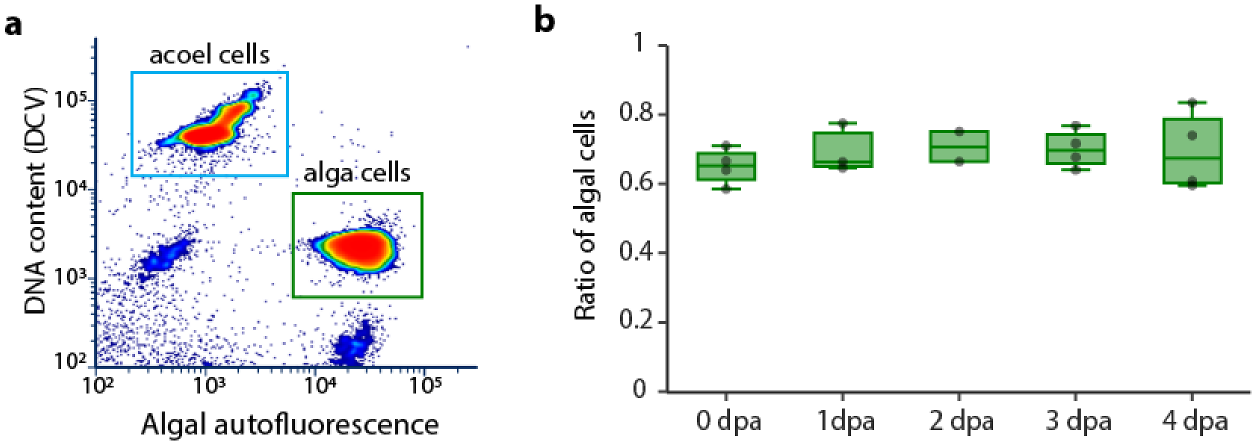

Supplementary Figure 2: Measuring the algal content using flow cytometry.
a Density plot showing cells after gating for singlets. Using the algal autofluorescence and DNA content (Dye Cycle Violet, DCV), we can identify acoel (cyan box) and algal (green box) events. **b** Quantification of algal content out of the total acoel and algal events. Boxes represent upper and lower quartiles with the median marked by the middle line, the bars represent the upper and lower fences. Individual replicates are shown as black dots. No significant difference between any samples was identified using an ANOVA test, $p = 0.86$. $n = 4$ biological replicates for each time point.

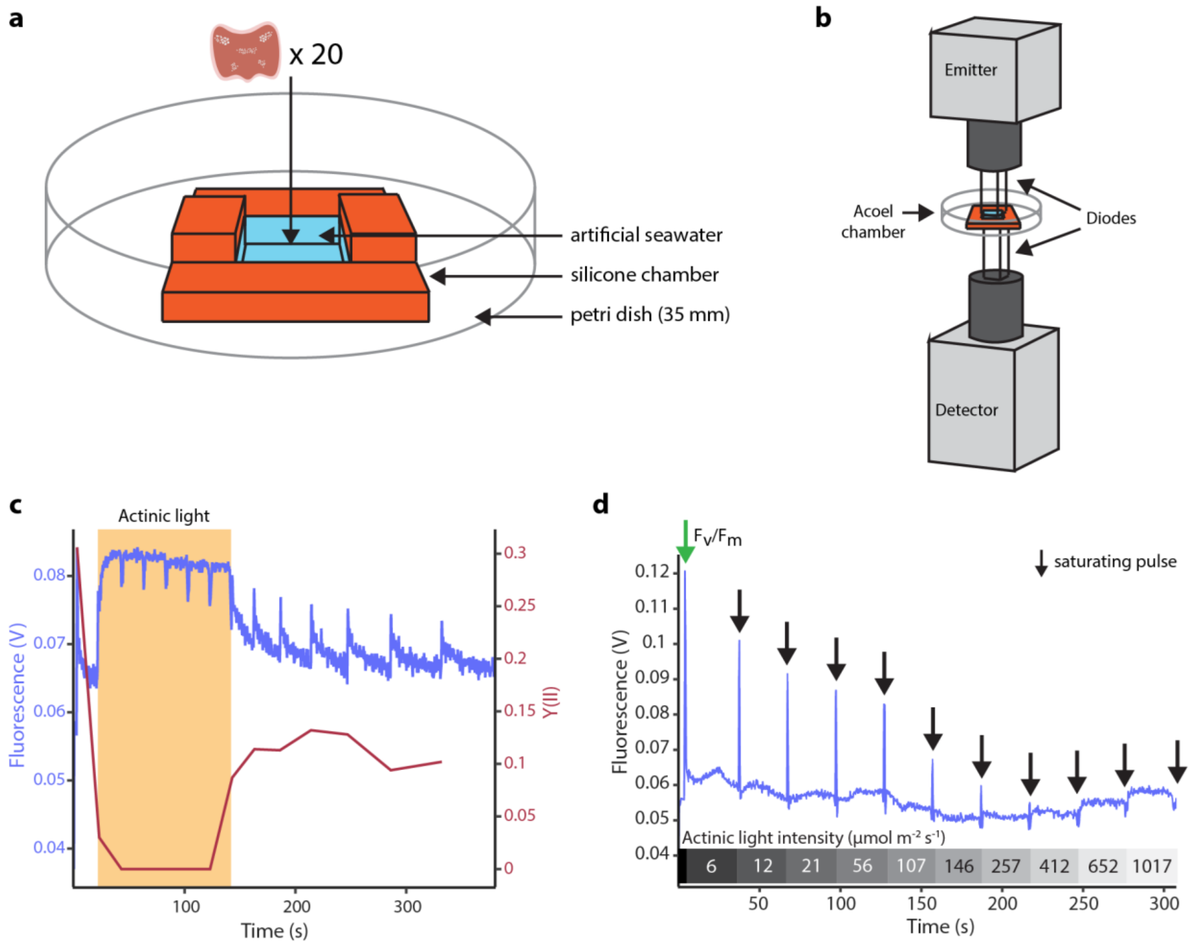

Supplementary Figure 3: Measuring photosynthetic efficiency of symbiotic algae within the acoel host using PAM fluorometry.

a Custom chamber designed for constraining acoels during PAM measurements. The chamber, made from a silicone mold, has an inner space of 5 x 6 x 2mm, and is mounted on a 35 mm petri dish. Two taller walls (4 mm in height) prevent the emitter diode from touching the water. For each experiment, 20 tails are transferred to the chamber right before measurements, and the chamber is filled by ASW. **b** The vertical setup of the PAM system is used to mount the acoel chamber between the emitter (top, covering the chamber's inner area) and the detector (bottom, in direct contact with the chamber).**c** Fluorescence measurement showing that DCMU treatment (20 µM) inhibits algal photosynthesis. After the first saturating pulse to measure F_v/F_m, a constant actinic light is turned on (orange background) causing the fluorescence to rapidly increase to the maximum level and the Y(II) to drop to zero, showcasing PSII blockage. Experiments were repeated for 4 times, each consisting of 10 whole animals. **d** Raw fluorescence trace of tails at 0 hpa. The bar at the bottom shows the actinic light intensity during the fluorescence measurements. Arrows: saturating pulses. The size of the fluorescence peak is used to calculate Y(II) at different actinic light intensities. Beyond ~150 µmol photons m^{-2} s^{-1}, Y(II) could not be calculated as the fluorescence signal under saturating pulses is indistinguishable from the background steady-state fluorescence. Experiment were repeated for 22 times with similar results.

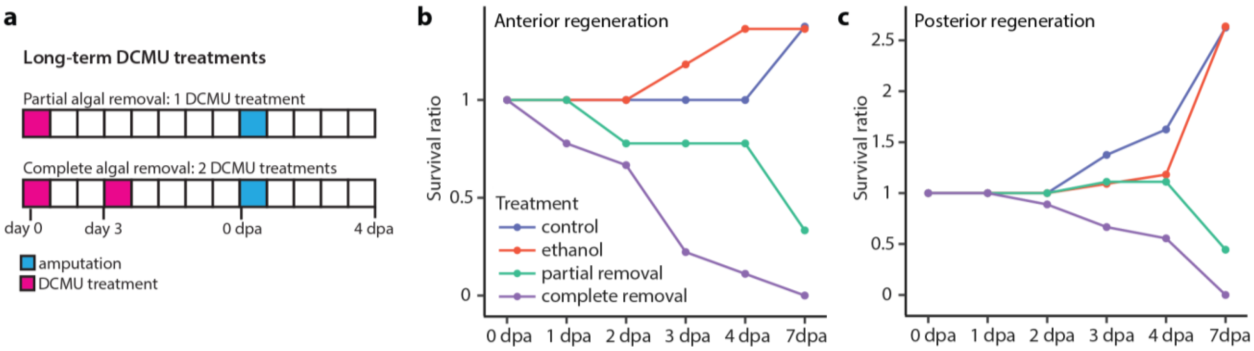

Supplementary Figure 4: Long term DCMU treatments lead to acoel death.
a Schematic showing the long-term DCMU treatment time course, with each square representing a day. The day of acoel amputation is marked in blue, while the days of DCMU treatments are marked in magenta. Each DCMU treatment lasts for 24 hr. A single DCMU treatment leads to partial algal removal, whereas two treatments are sufficient to remove algal cells completely. As DCMU is resuspended in ethanol, we treat animals with matching amounts of ethanol as controls.
b,c Survival ratio of animals from no-treatment control, ethanol-treated control, partial algal removal, and complete algal removal groups during anterior (**b**) and posterior (**c**) regeneration. The animals exhibited a range of regeneration capacities before death, which can be due to non-specific effects caused by the deteriorating animal physiology following algal depletion. In control groups, animals underwent fission, which resulted in more animals at the end of the experiment. n = 8 (control), 9 (partial removal, and complete removal), or 11 (ethanol) animals evaluated; the experiment was repeated twice.

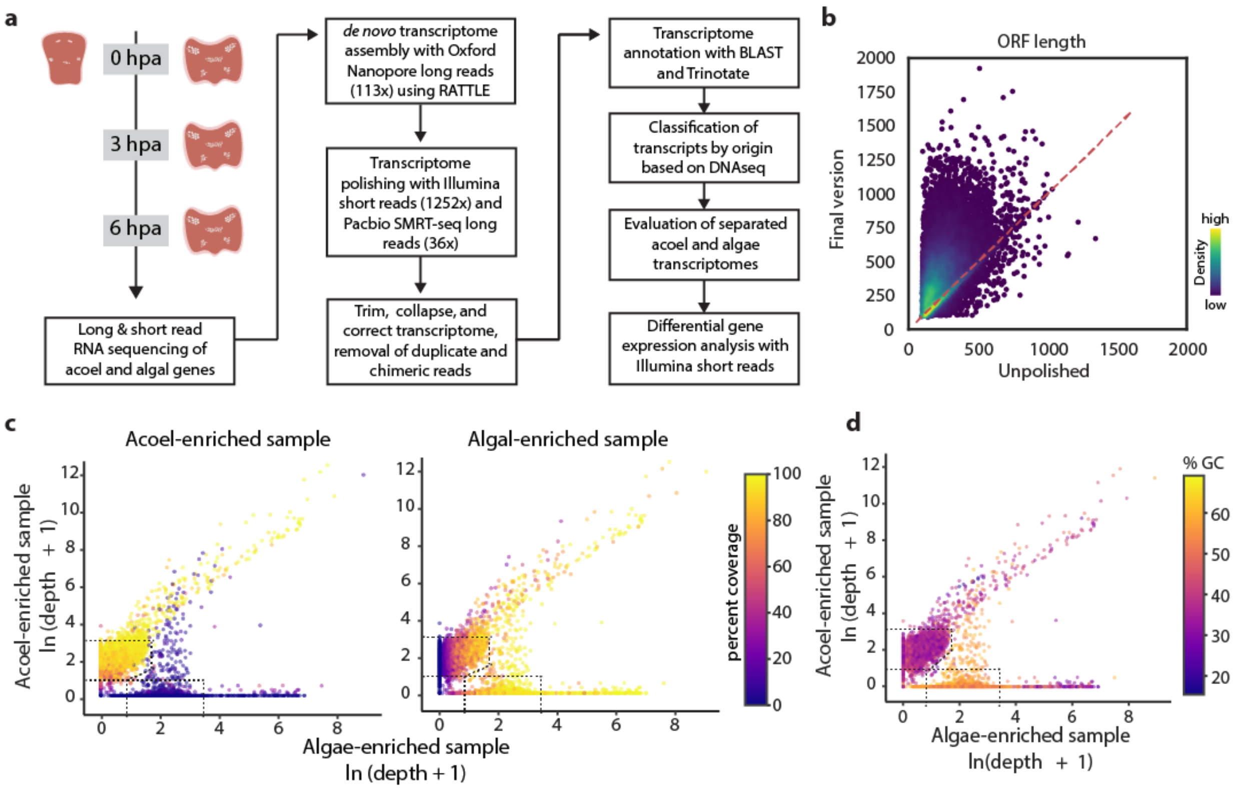

Supplementary Figure 5: *De novo* transcriptome assembly and annotation.

a Schematic of the workflow used to assemble the acoel and algal transcriptomes *de novo*. Schematics are created with BioRender.com.

b Density plot comparing ORF lengths before and after polishing. 77.8% of transcripts have longer ORF lengths after polishing.

c Percent coverage, the fraction of transcripts sampled by DNA reads of a particular sample, overlaid on the sequencing depth plot for the acoel-enriched (left) or alga-enriched samples (right).

d Percent GC content of each transcript overlaid on the sequencing depth plot. In general, algal transcripts have higher GC contents compared to acoel transcripts.

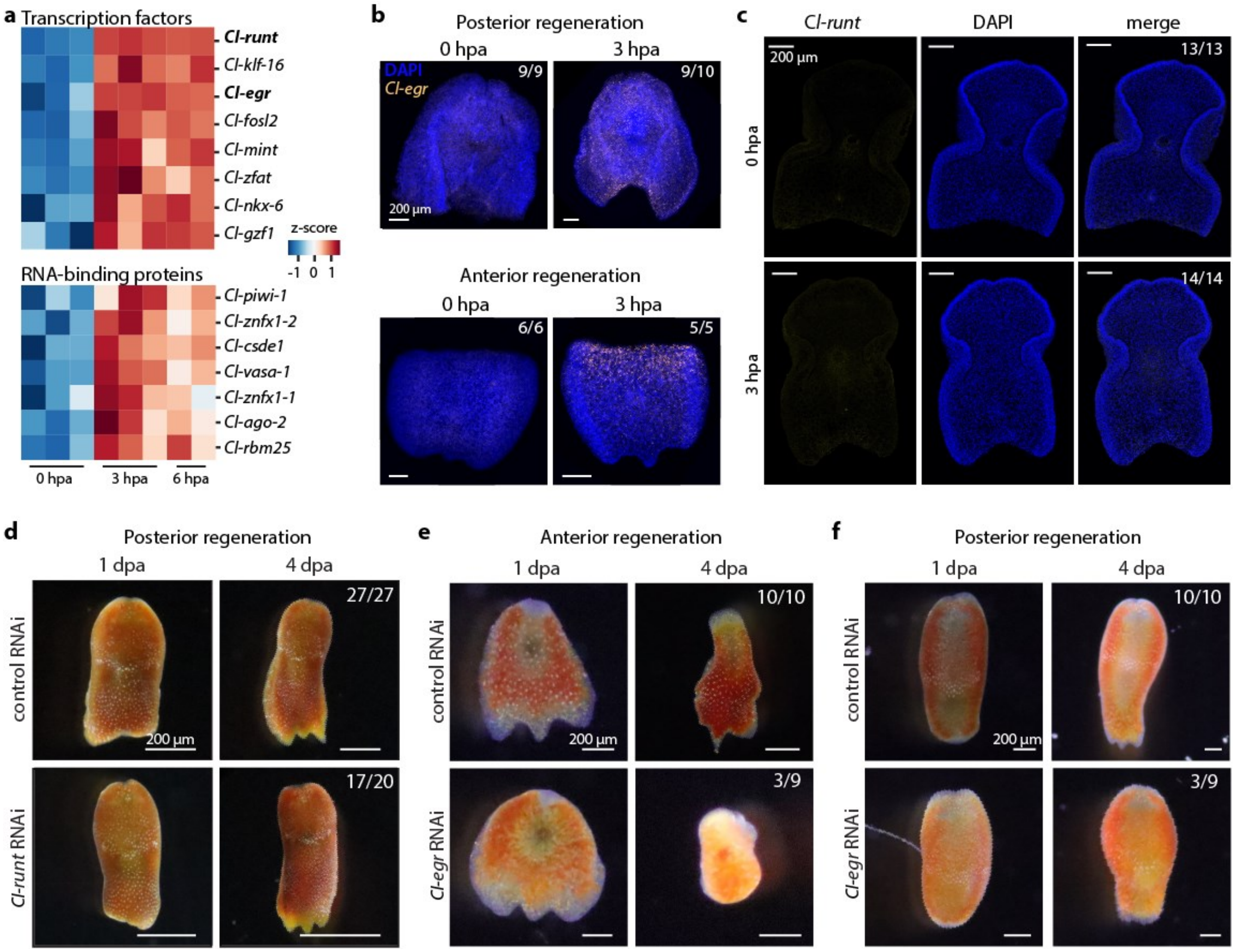

Supplementary Figure 6: Acoel wound responses.

a Heatmap of selected acoel regulatory genes upregulated after injury. Top: transcription factors; bottom: RNA-binding proteins.

b *Cl-egr* expression detected with fluorescence in situ hybridization (FISH) in heads and tails at 0 or 3 hpa.

c *Cl-runt* expression is not detected by hybridization chain reaction (HCR) in situ hybridization at 0 or 3 hpa at the posterior wounds.

d Most *Cl-runt* RNAi tail fragments regenerate three-lobed tails by 4 dpa. Numbers in (**d-f**) represent animals that regenerated similarly to the images shown out of the total evaluated animals.

e,f Approximately one-third of *Cl-egr* RNAi tail (**e**) and head (**f**) fragments fail to regenerate. Some tail fragments also experience tissue degradation making the phenotype difficult to interpret.

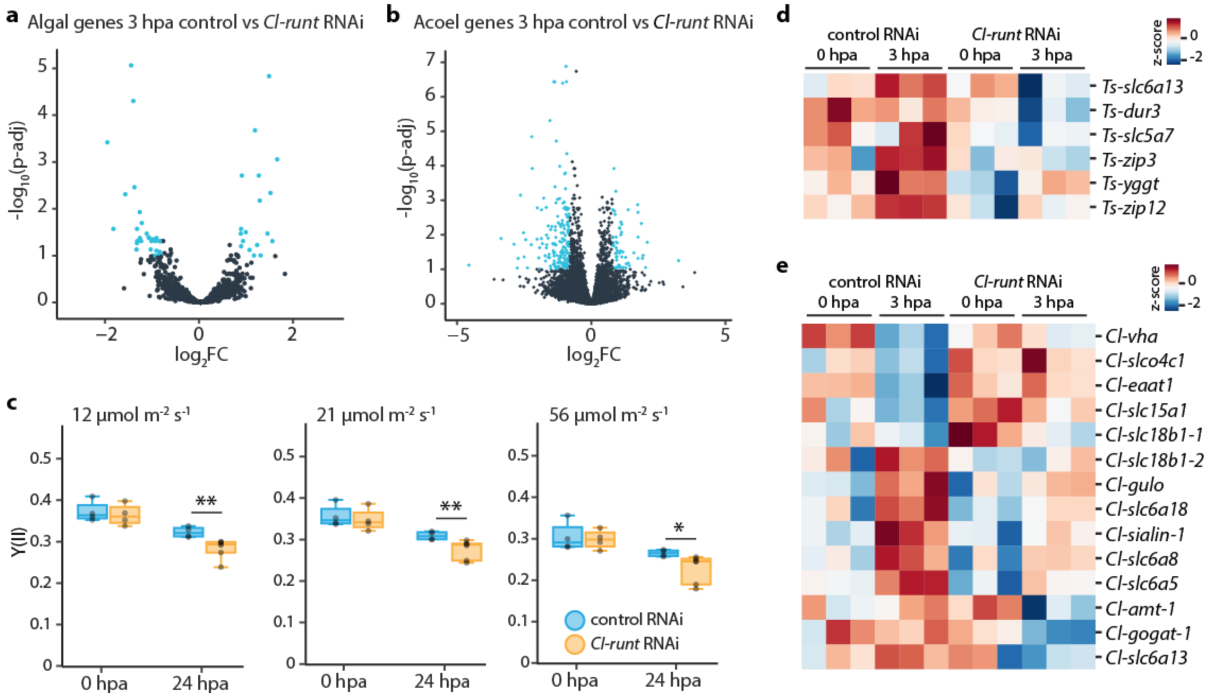

Supplementary Figure 7: Effects of *Cl-runt* RNAi on early regeneration responses.
a,b Volcano plots comparing algal (**a**) and acoel (**b**) genes at 3 hpa between control and *Cl-runt* RNAi samples. DEGs are shown in blue (log_2FoldChange ≧ 0.8 or ≦ -0.8, p-adj ≦ 0.1, calculated with DESEQ2 using Wald test followed by Benjamini and Hochberg correction).
c Y(II) of *Cl-runt* RNAi and control RNAi tails at 0 and 24 hpa with different intensities of actinic background light. Both *Cl-runt* RNAi and controls show a decrease in Y(II) at 24 hpa compared to their counterparts at 0 hpa. Y(II) of *Cl-runt* RNAi at 24 hpa compared to control RNAi is significantly lower (**p ≦ 0.05, *p ≦ 0.1; two-tailed t-test: 12 µmol photons m^{-2} s^{-1}, p = 0.017; 21 µmol photons m^{-2} s^{-1}, p = 0.031; 56 µmol photons m^{-2} s^{-1}, p = 0.067). Boxes represent upper and lower quartiles with the median marked by the middle line, the bars represent the upper and lower fences. Each treatment consisted of four replicates measured once, and each replicate contained twenty tails.
d,e Heatmaps of algal (**d**) and acoel (**e**) transporters affected by *Cl-runt* RNAi.

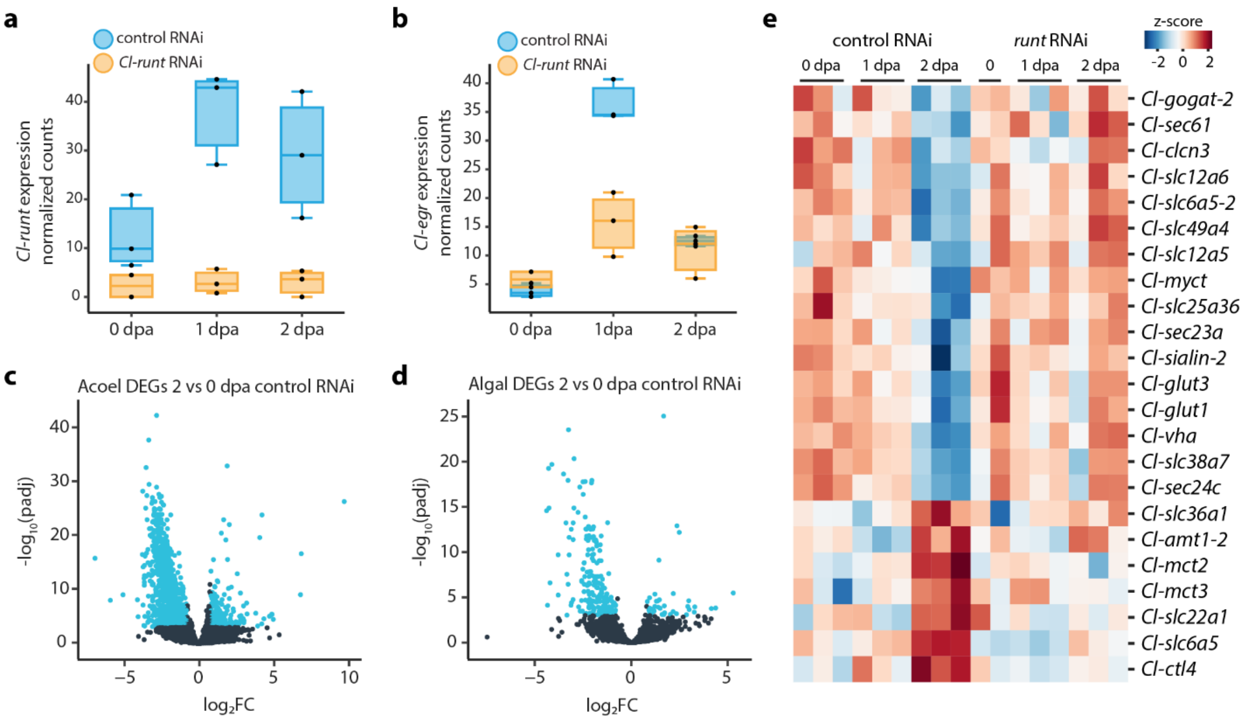

Supplementary Figure 8: Effects of *Cl-runt* RNAi on late regeneration responses

a,b Normalized expression of Cl-*runt* (**a**) and *Cl-egr* (**b)** at different days post amputation. Boxes represent upper and lower quartiles with the median marked by the middle line, with the bars representing the upper and lower fences. Black dots show values of individual replicates, n = 3 each consisting of 5 pooled acoels, per time point per treatment.

c,d Volcano plots comparing acoel (**c**) and algal (**d**) genes between 2 dpa and 0 dpa. DEGs are highlighted in blue (log_2FoldChange ≧ 0.8 or ≦ -0.8, p-adj ≦ 0.05, calculated with DESEQ2 using Wald test followed by Benjamini and Hochberg correction).

e Heatmap of acoel genes differentially expressed at 2 dpa in control RNAi animals. All genes shown are transporters except *Cl-gogat-2*.

Supplementary Tables

Supplementary Table 1. Summary of RNA sequencing runs for transcriptome assembly.

	Nanopore	Pacbio	Illumina
Number of raw reads	4,205,343	681,514	285,726,698
Number of filtered reads	3,224,900	679,170	280,739,473
Average read length (bp)	1,129	1,701	143
Transcriptome coverage	113x	36x	1,252x

Supplementary Table 2. Comparison of transcriptome quality. *H. miamia* transcriptome obtained from Gehrke, et al. [32], *Isodiametra pulchra* and *Tetraselmis suecica* obtained from NCBI database. The transcriptomes presented in this study have longer transcripts, fewer chimeric transcripts, and a higher fraction of transcripts with predicted ORFs.

	C. longifissura (this study)	*Tetraselmis sp.* (this study)	*H. miamia*	*Isodiametra pulchra*	*Tetraselmis suecica*
% complete + fragment BUSCOs	79.2	54.9	97.2	94.9	39.2
% transcripts without ORF	10.3	4.46	12.6	72.6	17.4
auN ORF length (aa)	519	491	753	882	554
auN transcript length (bp)	1,889	1,863	2,384	2,503	1,805
mean % GC	40.2	57.6	36.1	45.9	57.8
mean ORF length (aa)	359	366	345	121	319
mean transcript length (bp)	1,514	1,547	1,294	831	1,266
N50 ORF length (aa)	486	443	521	602	449
N50 transcript length (bp)	1,781	1,719	1,727	1,561	1,495
Number of transcripts	13,313	7,270	20,951	66,257	11,645
ORF auN/mean ratio	1.45	1.34	2.18	7.29	1.74
Total size (bp)	20,149,048	11,251,596	27,117,555	55,082,050	14,749,373
transcript auN/mean ratio	1.25	1.2	1.84	3.01	1.43
