# Supplementary material for: Coordinated wound responses in a regenerative animal-algal holobiont
Source: Nat Commun. 2024 May 13;15:4032. doi: 10.1038/s41467-024-48366-2 (PMC11091193; doi:10.1038/s41467-024-48366-2)
Supplement: Supplementary file 3 — Description of additional supplementary files [file 41467_2024_48366_MOESM3_ESM.pdf]

Description of Additional Supplementary Files

Supplementary Movie 1. Live imaging of algal movement around an anterior blastema at 2 dpa. Images are taken every 4 s for 5 min, time stamps in minutes:seconds. Scale bar, 50 µm.

Supplementary Movie 2. Live imaging of algal movement around a newly regenerated head at 3 dpa. Images are taken every 4 s for 5 min, time stamps in minutes:seconds. Scale bar, 50 µm.

Supplementary Data 1: Fasta file for the assembled acoel transcriptome.

Supplementary Data 2: Fasta file for the assembled algal transcriptome.

Supplementary Data 3. Gene annotations for acoel and alga transcriptomes. Species origin and gene names used in this study are provided in separate columns.

Supplementary Data 4. Oligo sequences used in this study. Oligo pools for HCR to detect Cl-runt expression, primers for cloning gene fragments used in RNAi (Cl-runt, Cl-egr) and ISH (Cl-egr, Cl-pc2) experiments, and custom oligos for RNA-seq experiments are listed with their sequences.

Supplementary Data 5: log2FoldChange and normalized counts of genes shown in the figures. Values are based on analysis with DESEQ2. Log2FC are only included if the padjusted values are smaller than or equal to 0.05.
