# Supplementary material for: Coordinated wound responses in a regenerative animal-algal holobiont
Source: Nat Commun. 2024 May 13;15:4032. doi: 10.1038/s41467-024-48366-2 (PMC11091193; doi:10.1038/s41467-024-48366-2)
Supplement: Supplementary file 11 — Reporting Summary [file 41467_2024_48366_MOESM11_ESM.pdf]

nature portfolio

Corresponding author(s): Bo Wang
James SIkes

Last updated by author(s): Mar 20, 2024

Reporting Summary

Nature Portfolio wishes to improve the reproducibility of the work that we publish. This form provides structure for consistency and transparency in reporting. For further information on Nature Portfolio policies, see our Editorial Policies and the Editorial Policy Checklist.

Statistics

For all statistical analyses, confirm that the following items are present in the figure legend, table legend, main text, or Methods section.

n/a	Confirmed	
☐	☒	The exact sample size (*n*) for each experimental group/condition, given as a discrete number and unit of measurement
☐	☒	A statement on whether measurements were taken from distinct samples or whether the same sample was measured repeatedly
☐	☒	The statistical test(s) used AND whether they are one- or two-sided<br>*Only common tests should be described solely by name; describe more complex techniques in the Methods section.*
☒	☐	A description of all covariates tested
☒	☐	A description of any assumptions or corrections, such as tests of normality and adjustment for multiple comparisons
☐	☒	A full description of the statistical parameters including central tendency (e.g. means) or other basic estimates (e.g. regression coefficient) AND variation (e.g. standard deviation) or associated estimates of uncertainty (e.g. confidence intervals)
☐	☒	For null hypothesis testing, the test statistic (e.g. *F*, *t*, *r*) with confidence intervals, effect sizes, degrees of freedom and *P* value noted<br>*Give P values as exact values whenever suitable.*
☒	☐	For Bayesian analysis, information on the choice of priors and Markov chain Monte Carlo settings
☒	☐	For hierarchical and complex designs, identification of the appropriate level for tests and full reporting of outcomes
☐	☒	Estimates of effect sizes (e.g. Cohen's *d*, Pearson's *r*), indicating how they were calculated

Our web collection on statistics for biologists contains articles on many of the points above.

Software and code

Policy information about availability of computer code

Data collection	python v. 3.9.7, plotly v. 5.3.1, pandas v. 1.3.4, numpy v. 1.20.3, seaborn v.0.11.2, scikit-posthocs v. 0.6.7, scipy v. 1.7.1, gseapy v. 1.0.4, matplotlib v. 3.4.3, Trimmmatic v. 0.39, nanofit v. 2.7.1, RATTLE, minimap2 v. 2.17 -r941, pilon v. 1.23, samtools v. 1.13, salmon v. 1.3.0, transdecoder v.5.5.0, trinotate v. 3.2.1, BUSCO v. 4.0.5, R version 4.2.2, DESeq2 v. 1.38.3, ggplot2 v.3.4.4, DualPam-100 Software
Data analysis	The transcriptome assembly and annotation pipeline are available at www.github.com/xuesoso/acoel_reference_assembly.

For manuscripts utilizing custom algorithms or software that are central to the research but not yet described in published literature, software must be made available to editors and reviewers. We strongly encourage code deposition in a community repository (e.g. GitHub). See the Nature Portfolio guidelines for submitting code & software for further information.

Data

Policy information about availability of data

All manuscripts must include a data availability statement. This statement should provide the following information, where applicable:

- Accession codes, unique identifiers, or web links for publicly available datasets
- A description of any restrictions on data availability
- For clinical datasets or third party data, please ensure that the statement adheres to our policy

The reference transcriptomes are available as Supplementary Data 1,2. The annotations are available in Supplementary Data 3. The RNA-seq data has been

deposited in the Gene Expression Omnibus (GEO) database under accession number GSE242841, and through SRA under the project number PRJNA1015130. The normalized read count and log2FoldChange values for all genes used in the figures are provided in Supplementary Data 5.

Research involving human participants, their data, or biological material

Policy information about studies with human participants or human data. See also policy information about sex, gender (identity/presentation), and sexual orientation and race, ethnicity and racism.

Reporting on sex and gender	N/A
Reporting on race, ethnicity, or other socially relevant groupings	N/A
Population characteristics	N/A
Recruitment	N/A
Ethics oversight	N/A

Note that full information on the approval of the study protocol must also be provided in the manuscript.

Field-specific reporting

Please select the one below that is the best fit for your research. If you are not sure, read the appropriate sections before making your selection.

☒ Life sciences ☐ Behavioural & social sciences ☐ Ecological, evolutionary & environmental sciences

For a reference copy of the document with all sections, see nature.com/documents/nr-reporting-summary-flat.pdf

Life sciences study design

All studies must disclose on these points even when the disclosure is negative.

Sample size	Sample sizes for RNA-seq experiments was determined based on minimum requirements for statistical power. Number of acoels used for PAM measurements was selected based on multiple iterations to optimize for strong and consistent fluorescence signal.
Data exclusions	Only one sample collected for Cl-runt RNAi 0 dpa (in the comparison with 1 and 2 dpa) was excluded since the amount of Cl-runt expressed was comparable to control RNAi samples, suggesting inefficient knockdown. For PAM measurements, data were excluded only if fluorescence traces were not discernable from background noise.
Replication	All experiments were repeated at least twice, on different dates, generating at least three independent biological replicates. For PAM measurements, animals were measured around the same time of day and only clear fluorescence traces were used for analysis. All experiments have biological and/or technical replicates. For RNAi experiments, regeneration was evaluated in both control and experimental groups. If the control RNAi animals did not regenerate, the experiment was discarded. For PAM analysis, biological replicates were discarded if the fluorescence was not distinguishable from the background fluorescence.
Randomization	All animals used were taken at the same time from the main colony and separated randomly into treatment groups.
Blinding	When evaluating the effects of knockdown on photosynthesis, researchers were blinded as to the specific gene knockdown.

Reporting for specific materials, systems and methods

We require information from authors about some types of materials, experimental systems and methods used in many studies. Here, indicate whether each material, system or method listed is relevant to your study. If you are not sure if a list item applies to your research, read the appropriate section before selecting a response.

Materials & experimental systems

n/a	Involved in the study
☐	☒ Antibodies
☒	☐ Eukaryotic cell lines
☒	☐ Palaeontology and archaeology
☐	☒ Animals and other organisms
☒	☐ Clinical data
☒	☐ Dual use research of concern
☒	☐ Plants

Methods

n/a	Involved in the study
☒	☐ ChIP-seq
☐	☒ Flow cytometry
☒	☐ MRI-based neuroimaging

Antibodies

Antibodies used

anti-BrdU monoclonal antibody (Sigma cat. #B2531)
FITC-conjugated goat anti-mouse secondary antibody (Sigma cat. #A6667)
anti-dig-POD (Roche, cat. # 11207750910)

Validation

These antibodies were previously used on other flatworms, and all experiments have tested multiple batches which generated consistent signals.

Animals and other research organisms

Policy information about studies involving animals; ARRIVE guidelines recommended for reporting animal research, and Sex and Gender in Research

Laboratory animals

Convolutriloba longifissura acoels, which are asexually reproducing through fission.

Wild animals

No wild animals were used

Reporting on sex

All animals are hermaphrodites

Field-collected samples

No samples were collected from the field

Ethics oversight

No ethical approval was required for working with these animals.

Note that full information on the approval of the study protocol must also be provided in the manuscript.

Plants

Seed stocks

Report on the source of all seed stocks or other plant material used. If applicable, state the seed stock centre and catalogue number. If plant specimens were collected from the field, describe the collection location, date and sampling procedures.

Novel plant genotypes

Describe the methods by which all novel plant genotypes were produced. This includes those generated by transgenic approaches, gene editing, chemical/radiation-based mutagenesis and hybridization. For transgenic lines, describe the transformation method, the number of independent lines analyzed and the generation upon which experiments were performed. For gene-edited lines, describe the editor used, the endogenous sequence targeted for editing, the targeting guide RNA sequence (if applicable) and how the editor was applied.

Authentication

Describe any authentication procedures for each seed stock used or novel genotype generated. Describe any experiments used to assess the effect of a mutation and, where applicable, how potential secondary effects (e.g. second site T-DNA insertions, mosiacism, off-target gene editing) were examined.

Flow Cytometry

Plots

Confirm that:

☒ The axis labels state the marker and fluorochrome used (e.g. CD4-FITC).

☒ The axis scales are clearly visible. Include numbers along axes only for bottom left plot of group (a 'group' is an analysis of identical markers).

☒ All plots are contour plots with outliers or pseudocolor plots.

☒ A numerical value for number of cells or percentage (with statistics) is provided.

Methodology

Sample preparation	Animals were dissociated on ice in the dissociation media (3.3× calcium magnesium free PBS, 2% FBS, 20 mM HEPES) by gently pipetting until solution was homogenized. The suspension was then filtered through a 40 µm strainer to remove debris and placed on ice. Cells were stained with 5 µM of Dye Cycle Violet (Invitrogen, cat. #V35002) for 20 min at room temperature. Before sorting, the solution was filtered again through a 35 µm strainer and gently mixed.
Instrument	Sony SH800S
Software	Sony SH800S software, FCS express 7
Cell population abundance	Algal cells were identified based on the low DNA content (Brilliant Dye Cycle Violet channel) and high algal autofluorescence (APC channel). Acoel cells were identified based on the high DNA content (Brilliant Dye Cycle Violet channel) and low algal autofluorescence (APC channel).
Gating strategy	Singlets were first gated based on FSC-A and FSC-H, and then algal and acoels cells were identified based on the DNA content (Brilliant Dye Cycle Violet channel) and algal autofluorescence (APC channel).

☒ Tick this box to confirm that a figure exemplifying the gating strategy is provided in the Supplementary Information.
